# Participatory research in health promotion: a critical review and illustration of rationales

**DOI:** 10.1093/heapro/daac016

**Published:** 2022-06-23

**Authors:** Janneke Harting, Kasper Kruithof, Lotte Ruijter, Karien Stronks

**Affiliations:** Department of Public and Occupation Health, Amsterdam UMC, University of Amsterdam, Amsterdam Public Health Research Institute, Meibergdreef 9, Amsterdam, 1105 AZ, The Netherlands; Department of Ethics, Amsterdam UMC, University of Amsterdam, Law & Humanities, Amsterdam Public Health Research Institute, Meibergdreef 9, Amsterdam, 1105 AZ, The Netherlands; Department of Public and Occupation Health, Amsterdam UMC, University of Amsterdam, Amsterdam Public Health Research Institute, Meibergdreef 9, Amsterdam, 1105 AZ, The Netherlands; Department of Public and Occupation Health, Amsterdam UMC, University of Amsterdam, Amsterdam Public Health Research Institute, Meibergdreef 9, Amsterdam, 1105 AZ, The Netherlands

**Keywords:** participation, research, review, case study

## Abstract

In health promotion research, enthusiasm for patient and public involvement (PPI) is growing. However, a lack of conceptual clarity leads to ambiguities in participatory processes and purposes, and hampers efforts to achieve and evaluate PPI in research. This study provides an overview of its underlying reasons—or rationales—so as to better understand, guide and interpret PPI in research practice. We conducted a critical review to identify typologies of rationales for PPI. We re-categorized the different types of rationales from these typologies based on their content. We illustrated the resulting categories of rationales with examples from a case study on PPI in research on Lyme disease. Five categories of rationales for PPI were identified. The democratic rationale reflects the normative right of citizens to have a voice in research. The consumerist rationale refers to the economic right of stakeholders with interests to have a say. Rooted in social justice, the transformative rationale seeks to empower marginalized groups. The substantive rationale starts from epistemic considerations and aims to improve the quality of knowledge that research generates. The instrumental rationale is of pragmatic origin and refers to improved efficiency and effectiveness of the research. Our overview of categories of rationales can be used as a frame of reference for PPI in health promotion research. Exploring, stating explicitly and reflecting on the underlying reasons for PPI may help to define realistic purposes, select matching approaches and design appropriate evaluation studies. This might also contribute to the conceptualization of PPI.

## INTRODUCTION

A recent position paper asserted that enthusiasm for patient and public involvement (PPI) in research has never been higher (McCoy *et al.*, 2018). PPI refers to incorporating the individual expertise of service users with lived experience and/or the collective perspectives of ‘ordinary’ citizens (Fredriksson and Tritter, 2017; Cook, 2021) in research-related activities and structures (Beresford and Russo, 2020; Cook, 2021). PPI is considered the key feature of participatory research (Cook, 2021). Participatory research covers a great variety of interrelated research approaches (Cornwall and Jewkes, 1995; Nitsch *et al.*, 2013), such as participatory evaluation, collaborative research and participatory action research (Cornwall and Jewkes, 1995; Cousins and Whitmore, 1998; Nitsch *et al.*, 2013). The popularity of PPI in research, which is also reflected by the requirements of research funders (McCoy *et al.*, 2018; Ball *et al.*, 2019), coincides with the growing attention for PPI in the broader social context (Ball *et al.*, 2019; Beresford and Russo, 2020). The anticipated gains of PPI include the relevance of the research, its efficiency and its effectiveness (McCoy *et al.*, 2018; Ball *et al.*, 2019).

While PPI is also becoming more common in health promotion research (Nitsch *et al.*, 2013), the evidence for the added value of PPI in research has been mostly summarized by review studies in other health domains. For example, PPI in health and social care research made service users feel empowered, valued and more skillful, while researchers gained a greater understanding of their research area (Brett *et al.*, 2014; Manafo *et al.*, 2018) and communities became more knowledgeable about their condition (Brett *et al.*, 2014). Another example is that PPI in health and health care research improved patient information, patient involvement in decision making and quality of care (Manafo *et al.*, 2018). It also increased study enrollment rates and aided researchers in securing funding, designing their studies and choosing relevant outcomes (Domecq *et al.*, 2014; Blackburn *et al.*, 2018; Crocker *et al.*, 2018). Despite these added values, reviews typically observed that evaluation studies of PPI in research usually poorly theorized the concept being studied (Brett *et al.*, 2010; Mockford *et al.*, 2012; Nitsch *et al.*, 2013; Lander *et al.*, 2014). The included studies rarely provided an explicit definition of PPI (Mockford *et al.*, 2012). Also, the studies typically lacked clear study objectives with respect to PPI (Lander *et al.*, 2014), started from policy frameworks rather than from models for PPI (Brett *et al.*, 2010; Mockford *et al.*, 2012; Lander *et al.*, 2014), and provided little detail about the process of the public involvement itself (Brett *et al.*, 2010; Nitsch *et al.*, 2013). Findings like these made the position paper conclude that a ‘striking lack of clarity’ remains about what PPI in research actually entails (McCoy *et al.*, 2018).

Central to its conceptualization are the underlying reasons—or rationales—for which PPI in research is set up (Boote *et al.*, 2002; McCoy *et al.*, 2018). We are aware of just one publication that touched upon such rationales for PPI in health promotion research (Nitsch *et al.*, 2013). In their review, the authors recognized two ‘streams’ of participatory evaluation (Cousins and Whitmore, 1998; Nitsch *et al.*, 2013). The first was practical participatory evaluation (Cousins and Whitmore, 1998; Nitsch *et al.*, 2013). Its underlying assumption—or rationale—is that PPI in research supports program, policy and organizational decision making (Cousins and Whitmore, 1998; Nitsch *et al.*, 2013) by enhancing the relevance, ownership and thus utilization of the evaluation findings (Cousins and Whitmore, 1998). The second stream was transformative participatory evaluation (Cousins and Whitmore, 1998; Nitsch *et al.*, 2013). Here, the underlying reason—or rationale—for PPI in research includes seeking to achieve social change by empowering members of community groups who are less powerful than, or are otherwise oppressed by, dominant groups (Cousins and Whitmore, 1998; Nitsch *et al.*, 2013). Despite the recognition of these two ‘streams’, the authors of the review concluded that, in health promotion research too, PPI has been prompted by situational and contextual factors rather than by theoretical definitions and distinctions (Nitsch *et al.*, 2013).

The lack of theoretical underpinning of PPI in research is problematic for two reasons. First, its hampers the implementation of PPI in research practice. While different rationales tend to be present—albeit mostly implicitly—in one and the same situation (Glimmerveen *et al.*, 2018; Schmidt *et al.*, 2020), dissimilar rationales are likely to become incompatible once they become manifest (Weaver and Cousins, 2007; Cornwall, 2008; Glimmerveen *et al.*, 2018). That is, the different reasons underlying PPI may have conflicting implications for the design of the participatory process, which includes dimensions like who to engage, i.e. which patients and/or public to involve, what to engage them in, in what way, and when (Weaver and Cousins, 2007; Ives *et al.*, 2013; Domecq *et al.*, 2014; Fredriksson and Tritter, 2017; Schmidt *et al.*, 2020). In other words, ‘[I]n many cases, recommendations about *how* the involvement should be undertaken cannot coherently be formulated without some sense of *why* it should be undertaken’ (McCoy *et al.*, 2018). The lack of clarity about what PPI entails may thus explain why it is frequently reported that expectations are not met (Blackburn *et al.*, 2018; Reed *et al.*, 2018) and that an overarching concern remains about tokenistic involvement (Domecq *et al.*, 2014).

Second, due to its poor conceptualization, evaluations of PPI in research have remained descriptive rather than evaluative (Brett *et al.*, 2010), so that the evidence base for its added value can be characterized as anecdotal, partial, weak and lacking coherence (Staniszewska *et al.*, 2011; Staley, 2015). This in turn also precludes further theory building (Brett *et al.*, 2010), for instance regarding the internal coherence of the concept of PPI (Brett *et al.*, 2010; Ives *et al.*, 2013), including how the different rationales for PPI link up with the various dimensions of the participatory process (Ives *et al.*, 2013; Nitsch *et al.*, 2013). Hence, it is necessary to reflect on and build an understanding of what PPI in research looks like in relation to the reasons for setting it up (Boote *et al.*, 2002; Nitsch *et al.*, 2013).

As we are not aware of any publication about the different underlying reasons for PPI in health promotion research, in this article, we provide a conceptual overview of rationales for PPI, with the ultimate aim of better understanding, guiding and interpreting PPI in health promotion research. In order to demonstrate the presence of different rationales for PPI in real-world research, as well as to support the interpretation of our review findings and to facilitate the identification and application of rationales, we will illustrate each of the presented rationales with a practical example from a case study on patient involvement in a research program on Lyme disease in the Netherlands.

## METHODS

### Critical review

We used a critical review methodology, as such a methodology aims to identify relevant publications, is appropriate for arriving at a synthesis of existing schools of thought, and—instead of starting from a hypothesis—typically results in a conceptual model (Grant and Booth, 2009). Here, the model consists of conceptually distinct categories of rationales for PPI in health promotion research. Our critical review followed the Search, Appraisal, Synthesis and Analysis (SALSA) framework (Grant and Booth, 2009).

#### Search

With our search, we aimed to gain as complete as possible a picture of prevailing rationales for PPI. Therefore, we searched for reasons underlying PPI in research, policy and practice, in both the health promotion domain and other domains. First, we examined the literature compiled during our previous work on PPI in health promotion (Fienieg *et al.*, 2008, 2012; Harting and Van Assema, 2011, 2017). Second, we searched PubMed and PsycINFO for publications with the following terms, as well as related terms, in the title and/or the abstract: (a) ‘motives’, ‘reasons’ or ‘rationales’; and (b) ‘involvement’, ‘engagement’ or ‘participation’; and (c) ‘health’, ‘health promotion’ or ‘research’. We did not limit the publication date, as publications explicitly reporting on rationales appeared to be scarce. Third, we scanned papers thought to be relevant for the actual presence of rationales for PPI. Fourth, we applied extensive snowballing and forward citation searching to papers including such rationales. The search strategy was first carried out in 2015, and extensively updated in 2021. Finally, JH selected 47 publications for further appraisal.

#### Appraisal

As is common for this type of review (Grant and Booth, 2009), we did not undertake a formal quality assessment. Instead, JH and KK examined and discussed each publication for its conceptual contribution. Documents that, according to both JH and KK, included a theory-driven or empirically derived typology of rationales for PPI were selected for further synthesis (*n* = 28). Excluded were publications that merely referred to or duplicated previously described typologies of rationales, focused on dimensions or determinants for involvement, lacked a sufficiently detailed description or did not make a conceptual contribution (*n* = 19; references in Supplementary File S1).

#### Synthesis

A critical review, such as ours, usually uses a narrative synthesis (Grant and Booth, 2009). From the 28 included papers, JH extracted the following information: (a) authors, year and type of publication, and domain and type of participants; (b) the original names given to the rationales and a summary of all the rationales for PPI described. This resulted in 23 typologies of rationales for PPI (Supplementary File S2): in research in the health domain (*n* = 5), in research in a domain other than health (*n* = 4), in policy or practice in the health domain (*n* = 9), and in policy or practice in a domain other than health (*n* = 5). A comparison of the typologies by JH and KK revealed that authors differed in the number of rationales they distinguished, as well as that different names could be used for rationales with a similar content, and that similar names could be attached to rationales with a different content. Therefore, in order to arrive at conceptually distinct categories of rationales for PPI, we chose to base our narrative synthesis on a content-driven analysis (Popay *et al.*, 2006; Grant and Booth, 2009).

#### Analysis

From the summaries of rationales, JH and KK defined distinct categories of rationales based on the characteristics that were regarded as most discriminative, i.e. those that were most likely to typify one category, but least likely to typify any of the other categories. Names for each of the categories were derived from the original publications by choosing the corresponding name that was regarded as the most descriptive. The resulting five conceptually different categories of rationales were reviewed and agreed upon by all authors.

### Illustrations

#### Participatory case

Practical examples to illustrate the rationales for PPI were selected from a case study on patient involvement in research on Lyme disease (2017–2018). This qualitative study was inspired by the findings of the literature search we conducted in 2015. The participation of patients was organized as part of a special Lyme research program by the Netherlands Organization for Health research and Development (referred to below as the funder; ZonMw, 2015). The research program, worth 1 million euros, was commissioned by the Dutch Minister of Health, Welfare and Sports in response to a debate in the Lower House of the Dutch parliament about a citizens’ initiative’s petition asking for more research on Lyme disease. A ministerial requirement for the program was the involvement of the two Dutch patient associations for Lyme disease.

The call for research proposals stated that: ‘The emphasis lies on research that meets the questions that address the knowledge gap experienced by Lyme patients in order to build a bridge between patients, practitioners and scientists’ (ZonMw, 2015). This bridge was thought to be needed to solve the heated dispute between patients and researchers about the case definition of Lyme disease. Patients with chronic symptoms in particular strongly disagreed with the latest guidelines (CBO, 2013), as the approved diagnostic tests would mean that chronic patients like them did not have the disease. A simultaneous aim of the research program was to set the national agenda for Lyme research (ZonMw, 2015).

The involvement of patients included taking part in two invitational conferences to decide on the research agenda for the Lyme program; assessing the initial proposals for research (first assessment); taking part in the design of the full study proposals; assessing the full research proposals (second assessment); staying connected to the studies—for which the grants had been awarded—while they were being conducted; and playing a part in the interpretation of the research findings in the scientific reports.

#### Interviews

To learn how the patient involvement took shape, we asked the 12 persons who had a key role in the participatory process to share their experiences. LR conducted 10 semi-structured interviews with representatives of the patients’ associations (*n* = 2), the principal researchers of the studies (*n* = 3), staff members of the funder (*n* = 3; group interview), the trainer who supported the patients (*n* = 1), and members of the assessment committee of the research program (*n* = 3; one of whom had chaired the two invitational conferences on the Lyme research agenda). Perceptions discussed included the underlying reasons for participation (i.e. the rationales), the design of the participatory approach (i.e. dimensions such as which patients were involved, in what, how and when), the course of the participatory process, and the type of results that the participation was yielding.

The interviews were transcribed verbatim. The transcripts were analyzed for manifestations of the different rationales for PPI by JH, KK and LR. The findings from this analysis induced us to update our literature review in 2021. The qualitative analysis was mostly deductive, as we aimed to identify the theoretical concepts, i.e. the rationales for PPI, in the interview data. Examples to illustrate the different rationales were discussed by JH and KK until consensus was reached.

#### Ethical considerations

According to the Dutch Medical Research Involving Human Subjects Act, this study did not require approval by a medical research ethics committee.

All 12 respondents gave verbal consent for the use of the interview data for research purposes. Two of them additionally wanted to read and give permission for the publication of quotations from their interview. Quotations of seven different respondents were used to illustrate the presence of the different rationales in the Lyme research case. To warrant their anonymity, the respondents are referred to only by their role in the research program, e.g. representative of patient association, researcher or funder.

## RESULTS

### Categories of rationales

The five categories of rationales we distinguished were democratic (in 19 typologies), consumerist (in 5), transformative (in 15), substantive (in 12) and instrumental (in 20). The democratic rationale is in essence deontological (PPI as an end in itself), while the substantive and instrumental rationales are of consequentialist origins (PPI as a means to an end). The consumerist and transformative rationales both include deontological and consequentialist elements.

#### Democratic

In general, the democratic rationale highlights the normative right of citizens to democratic decision making (Stirling, 2006, 2008; Knaapen and Lehoux, 2016). A participatory process is seen as self-evidently good, without reference to the ends in question (Stirling, 2008). All interested or affected citizens should be represented in the assessment and selection of alternatives (Charles and DeMaio, 1993; Stirling, 2006, 2008). Democratic decision making additionally reflects the desirability of equity of access, empowerment of process and equality of outcomes for all citizens (Stirling, 2006, 2008). Although in principle an end in itself, democracy is also described as a means to protect citizens from decisions going against their interests (Conklin, Morris, and Nolte, 2010) and to counter the prevailing powers (Wesselink *et al.*, 2011).

In the context of research, the democratic rationale refers to the right of citizens to have a voice in research that may affect them (Cousins and Whitmore, 1998; Boote *et al.*, 2002; Weaver and Cousins, 2007; Gradinger *et al.*, 2013). This implies that PPI in research is the right thing to do and is of intrinsic value (Gradinger *et al.*, 2013; Ball *et al.*, 2019). Citizens should have a voice in the formulating, conducting and implementing of research (Mielke *et al.*, 2016; Schmidt *et al.*, 2020). Through the representation of their values and preferences, PPI contributes to transparent, accountable and responsible research (Gradinger *et al.*, 2013; Ives *et al.*, 2013). In research too, democracy is described as a means to an end: it could equalize power imbalances between the public and the academic community (Ives *et al.*, 2013).

The presence of the democratic rationale in the Lyme research case is illustrated in Box 1, suggesting that the issue of representation might be important in the ‘who’ dimension.

**Box 1: Illustration of the democratic rationale**

The normative right of Lyme patients to democratic decision making was expressed by the chair of the invitational conferences:

In a way you can now no longer conduct research without involving the target group in it. [_**…**_] The usual norm in our society is that of patient participation.

[Chair of invitational conferences]

Giving Lyme patients a voice in the research about the disputed case definition of Lyme disease was broadly approved, but it was contested whether merely involving patients also incorporated democratic decision making in the national research agenda for Lyme disease.

Well I think that, as such, [the involvement of Lyme patients] has been a good initiative. [_**…**_] But saying that what was discussed [at the invitational conferences] will from now on constitute the national Lyme disease research agenda is of course *slightly* odd.

[Researcher]

This researcher’s objection was that Lyme patients were too limited a group of citizens to decide on the public health issue that the researcher thinks Lyme disease is. This opinion was supported by the reference made to the democratic rationale by a member of the program committee.

Civilians do think very differently than patients do. [_**…**_] If you should ask someone from the general public, so a civilian who doesn’t yet have the disease, how you can avoid getting [Lyme disease]? For a civilian that is a relevant question.

[Member of the program committee]

The researcher—implicitly—agreed that a more comprehensive democratic approach would do more justice to the complexity of Lyme disease and the broader research scope needed to tackle it effectively.

If I’m right the word prevention is not mentioned in [the funder’s] research programme. How is that possible? How on earth is that possible? [_**…**_] As a researcher, I can’t understand that at all! But I do understand why this happened. [_**…**_] What use is a vaccine to people who currently have Lyme disease? That doesn’t cure today’s Lyme patient. So I can understand that from the patients’ point of view.

[Researcher]

#### Consumerist

Consumerist justifications for citizen involvement relate to neo-liberal and economic rights (Boote *et al.*, 2002), such as the right to individual choices in the marketplace (Tritter and McCallum, 2006) and the rights of individual taxpayers or consumers in health care (Tritter and McCallum, 2006; Knaapen and Lehoux, 2016). Consumer involvement is an essential component of markets (Tritter and McCallum, 2006): it strengthens the public’s voice in decisions about the organization and delivery of health services (Tritter and McCallum, 2006). As a means to an end, it may also promote patient-focused care (Tritter and McCallum, 2006), by incorporating patients’ personal preferences and decision making in health care (Knaapen and Lehoux, 2016). Consumers or customers may demand services to be the way they want them, which in turn may also influence service outcomes (Conklin *et al.*, 2010). Involvement is then a lever to enhance the responsiveness of service providers (Knaapen and Lehoux, 2016) and to redress power inequalities between health professionals and patients (Tritter and McCallum, 2006).

In research, the consumerist rationale includes value-for-money justifications (Boote *et al.*, 2002) similar to those explained above. It particularly concerns the rights of stakeholders with—individual, organizational or political—interests (Mielke *et al.*, 2016), such as lobby groups (Mielke *et al.*, 2016), to have a say in and demand what they consider the best research. Involvement may then serve as a tool for both scientists and societal groups to advocate their perceptions and values (Mielke *et al.*, 2016), so as to ensure that the studies conducted and/or the knowledge produced will support—or at least not conflict with—their interests.

The presence of the consumerist rationale in the Lyme research case is illustrated in Box 2, indicating that patients and researchers might represent opposing stakeholder groups.

**Box 2: Illustration of the consumerist rationale**

Accounts of the run-up to the research program and the invitational conferences reflected a consumerist rationale, with patients and researchers as opposing stakeholder groups promoting their respective interests and imposing their perceptions on each other. That is, the patients entered ‘the market place’ with predefined issues stated in their petition.

Our petition consisted of eight points, [for instance] about better registration of Lyme patients, in-service training of doctors, development of better tests, more research into chronic Lyme disease, more attention to the opportunities for treating chronic Lyme disease [_**…**_].

[Representative of patient association]

Against these consumer rights, the researchers raised and defended their ‘undisputed’ research findings.

What struck me was [that these] rather deviant opinions about the interpretation scientific research were raised very forcefully. [_**…**_] That some from the patient movement very definitely rejected certain scientific research findings. [_**…**_] And they include examples where I think well, if you just really brush aside the outcomes of well-designed scientific research [_**…**_] well, then it sometimes becomes a challenge to keep up a useful discussion. [_**…**_] That is then often part of the final 10 percent that you can’t agree on.

[Researcher]

#### Transformative

In general, the transformative rationale is rooted in the normative concept of social justice (Cousins and Whitmore, 1998; Taylor *et al.*, 2008; Preston *et al.*, 2010). Core to this rationale is the amelioration of social inequities (Cousins and Whitmore, 1998). Providing oppressed and marginalized groups with opportunities to have a voice (Cousins and Whitmore, 1998) allows them to gain control over the decisions that affect their lives (Cornwall, 2008; Fienieg *et al.*, 2008; Taylor *et al.*, 2008; Preston *et al.*, 2010). Such transformation links up to the notion of empowerment (White, 1996; Cornwall, 2008), which may serve both as a means and an end (White, 1996). Empowerment encompasses awareness raising, social learning and capacity building (Parry *et al.*, 1992; Litva *et al.*, 2002; Schmidt *et al.*, 2020; McCarron *et al.*, 2021).

For individuals, empowerment may strengthen competencies, responsibility (Parry *et al.*, 1992; Litva *et al.*, 2002) and political consciousness and engagement (Oakley, 1989; Conklin *et al.*, 2010; Head, 2011). For communities, empowerment relates to intensified integration, trust, citizenship and democracy (Parry *et al.*, 1992; Litva *et al.*, 2002; Head, 2011) and to the reinforcement of structures, networks and health promotion efforts (Cornwall, 2008; Fienieg *et al.*, 2008). For participants, involvement may also provide opportunities to express political identity and belonging (Parry *et al.*, 1992; Conklin *et al.*, 2010).

In research, PPI conforming to the transformative rationale has the potential to strengthen disadvantaged and disempowered groups by giving them a chance to speak out on research issues (Boote *et al.*, 2002). Transformative involvement seeks to overcome discrimination and oppression (Gradinger *et al.*, 2013), to ameliorate social inequities (Cousins and Whitmore, 1998; Weaver and Cousins, 2007) and to promote fairness, emancipation and empowerment (Cousins and Whitmore, 1998; Webler and Tuler, 2006; Weaver and Cousins, 2007; Schmidt *et al.*, 2020). This may require negotiating a balance between developing valid generalizable knowledge and benefiting the community (Boote *et al.*, 2002), e.g. through egalitarian deliberation (Webler and Tuler, 2006).

The presence of the transformative rationale in the Lyme research case is illustrated in Box 3, suggesting that this rationale was endorsed by both the funder and the patient associations.

**Box 3: Illustration of the transformative rationale**

From the perspective of the funder, the rationale for patient participation in the research program was transformative. The funder expressed—implicitly—the opinion that chronic Lyme patients, who had been closely involved in drafting the current case definition of Lyme disease, were ‘wrongfully’ excluded from having the disease by that very definition. Hence, for the funder, the aim of patient involvement was to do them justice by taking them more seriously.

We are now going to draw up the research agenda, and what is important for us now is to identify the problems that are currently causing the undesirable situation in practice. In the end, we want to achieve a situation in which patients are correctly recognized and identified. That problems are taken seriously, that they get appropriate treatment.

[Staff member of funder]

Here, chronic Lyme patients are portrayed as a marginalized group for which fairness is promoted, and patients indeed felt supported by the funder’s actions in line with this transformative starting point.

So we then went and talked to [the funder]. And then we were listened to. [_**…**_] And *[*the funder**]** took that seriously and very_**…**_ [The funder] took a step back to be able to do so. And that process was addressed very carefully, to get everyone around the table. [_**…**_] to see whether it was possible to start a dialogue.

[Representative of patient association]

#### Substantive

In general, the substantive rationale is based upon epistemic considerations, i.e. regarding theories of knowledge (Cousins and Whitmore, 1998; Weaver and Cousins, 2007). Substantive involvement seeks the inclusion of more diverse, extensive and context-specific bodies of knowledge, in order to increase the quality of the information underlying decision making (Stirling, 2006, 2008; Wesselink *et al.*, 2011). It assumes that non-experts see problems, issues and solutions that experts miss (Wesselink *et al.*, 2011). The resulting higher quality of information may also improve the quality of the decisions themselves (Stirling, 2006, 2008).

In a research context, substantive PPI refers to the inclusion of lay theories, experiential knowledge, local knowledge and the importance of context (Cousins and Whitmore, 1998; Boote *et al.*, 2002; Weaver and Cousins, 2007; Knaapen and Lehoux, 2016), with the aim to improve the quality of knowledge as the main research output (Boote *et al.*, 2002; Gradinger *et al.*, 2013; Ives *et al.*, 2013; Ball *et al.*, 2019; Schmidt *et al.*, 2020). By adding such insights to the design, methods and findings of the research (Ives *et al.*, 2013), substantive PPI produces new and more reliable, credible and valid knowledge (Cousins and Whitmore, 1998; Boote *et al.*, 2002; Weaver and Cousins, 2007; Gradinger *et al.*, 2013; Ives *et al.*, 2013). The exchange between—and integration of—various perspectives, approaches and bodies of knowledge, one of which is scientific knowledge (Schmidt *et al.*, 2020), produces valid representations of social phenomena (Cousins and Whitmore, 1998; Weaver and Cousins, 2007) and a socially robust, holistic and shared understanding of research problems and objectives (Schmidt *et al.*, 2020). Giving lay participants a say in the formulation of research questions promotes relevant research which is meaningful to the communities affected by the issue being studied (Boote *et al.*, 2002; Gradinger *et al.*, 2013; McCarron *et al.*, 2021).

The presence of the substantive rationale in the Lyme research case is illustrated in Box 4, showing how knowledge integration occurred in the design phase of Lyme studies.

**Box 4: Illustration of the substantive rationale**

The substantive rationale was reflected by—among other things—substantial changes made to the research proposals, which reflected the integration of patients’ experiential knowledge with scientific knowledge. One example was the inclusion of Lyme patients who did not meet the prevailing case definition for the disease.

We really changed the study design to accommodate this. [_**…**_] We hadn’t done so based on our initial line of approach, as we thought well look, we want to study Lyme disease, but we need to be sure that these people do have Lyme. [_**…**_] And looking back on it I think we were right not to stick to this, and also include patients who could not be said with certainty to have Lyme disease, but who were suspected of having it.

[Researcher]

Another example of the integration of patients’ experiential knowledge was the adoption of an additional diagnostic test, which was regarded by the researchers as not yet validated, and which had so far not been released by its producer for further validation study.

It did work out well, as we’re still trying to, we’re going to examine some tests and we already have three [valid tests], and we’re trying the fourth [not yet validated] one. And by having the patients involved, it turns out to create some momentum that will enable us to also include this fourth test. So that’s funny in a way. So that’s what I thought afterwards, as I was a bit apprehensive about it at first.

[Researcher]

#### Instrumental

In general, the instrumental rationale refers to involvement as a means of achieving particular goals, such as health and wellbeing (Taylor *et al.*, 2008; Preston *et al.*, 2010) by increasing the efficiency and/or effectiveness of policies, programs and services in health or health promotion (Oakley, 1989; Charles and DeMaio, 1993; Stirling, 2006; Fienieg *et al.*, 2008). The policy goals themselves are not open to discussion (Stirling, 2008; Wesselink *et al.*, 2011). Instrumental involvement may also contribute to cost-effectiveness through the use of community resources (Oakley, 1989; Morgan, 2001; Cornwall, 2008), meaning it may involve costs for citizens (White, 1996; Cornwall, 2008).

In research, the instrumental rationale offers a pragmatic justification for PPI (Cousins and Whitmore, 1998; Weaver and Cousins, 2007), with the involvement serving as a means to an end (Ives *et al.*, 2013). In terms of effectiveness, instrumental PPI makes science more sensitive to societal problems (Mielke *et al.*, 2016), which increases the usefulness of the knowledge created (Cousins and Whitmore, 1998; Weaver and Cousins, 2007). It also increases the likelihood that changes take place (Mielke *et al.*, 2016) and real progress is made on the problem under investigation (Webler and Tuler, 2006). In terms of efficiency, instrumental PPI increases the acceptability, legitimacy and ownership of the research process, its outcomes and the solutions of problems studied (Ball *et al.*, 2019; Schmidt *et al.*, 2020). Therefore, it also enhances the legitimacy for an agency to act upon the study findings (Webler and Tuler, 2006).

In research practice, instrumental PPI can support the recruitment of peers (Boote *et al.*, 2002; Ball *et al.*, 2019; McCarron *et al.*, 2021), the access to marginalized groups (Boote *et al.*, 2002) and the retention of participants in studies (Ball *et al.*, 2019). It may include the co-building of frameworks, tools and strategies (McCarron *et al.*, 2021), and the improvement of other research products (Ball *et al.*, 2019). Finally, lay participants may provide the motivation and capacity to disseminate research information to peers (Boote *et al.*, 2002), and to assist the implementation (Gradinger *et al.*, 2013) and dissemination of research finding (Ives *et al.*, 2013).

The presence of the instrumental rationale in the Lyme research case is illustrated in Box 5, showing how patient contributed to the feasibility and efficiency of Lyme studies.

**Box 5: Illustration of the instrumental rationale**

The instrumental rationale became manifest in the patients’ involvement in the design and the conduct of the studies. The first example, about the burden to patients of participating in the study, relates to the study’s feasibility.

Sometimes just very practical things. [_**…**_] I think with the proposal about this PET scan they had the idea that they wanted to do everything in one day at the intake. So then the patients said, well, isn’t that a bit too burdensome for these Lyme patients. [_**…**_] It sounds great, but [*..*.] you might have to divide it over two visits, or whatever.

[Representative of patient association]

The second example, about speeding up the inclusion of patients, primarily refers to the efficiency of the research process, while it may ultimately also help improve the study’s effectiveness.

Of course we’re still starting up the study. So we were able to brainstorm with the patients about how we want to facilitate the inclusion further, and it’s always good to be able to discuss that with them.

[Researcher]

Currently they’re having trouble recruiting enough patients during the first six months. Actively contributing ideas about how we can communicate everything through the patient society. To get people to join the study sooner.

[Representative of patient association]

## DISCUSSION

### Summary

In our critical review, we identified five categories of underlying reasons for PPI in research. The democratic rationale reflects the normative right of citizens to have a voice in research that affects them. The consumerist rationale refers to the economic right of stakeholders with interests to have a say in and demand the best research. Rooted in social justice, the transformative rationale seeks to empower marginalized groups by giving them a say in research. The substantive rationale is based on epistemic considerations and aims to improve the quality of knowledge that research generates. The instrumental rationale is of a pragmatic origin and refers to improved efficiency and effectiveness of the research. The practical examples from a case study on PPI in a research program on Lyme disease in the Netherlands demonstrated the simultaneous presence of all five rationales.

### Limitations

The first limitation of our overview of rationales is that we largely followed the classifications made by the original authors. As a result, the categories we distinguished still have overlapping features, i.e. the—in principle—rights-based rationales (PPI as an end in itself) also include consequences that link up with the substantive or instrumental rationales (PPI as a means to an end). Alternatively, we could have reduced each category to its core, by splitting up the original definitions and by allocating the different parts to these most discriminating characteristics. This could also have led to subcategories, such as individual and community empowerment as part of the transformative rationale. Such more sharply defined categories could yield even more clarity about the underlying reasons for using PPI in research. Further refinement procedures should preferably include research groups from a variety of scientific domains, as we saw how different domains identified different rationales in their typologies (Supplementary File S2). In our experience, agreed-upon criteria for the intended core rationales might be essential, although it may still require discussion to apply these criteria consistently.

The second limitation is that our categories of rationales may not be complete. As critical reviews are usually not as systematic as other types of reviews (Grant and Booth, 2009), we may have overlooked relevant publications on typologies of rationales for PPI. Nor can we exclude the possibility that other researchers would have made different decisions on which papers to include in—or exclude from—the overview of typologies of rationales (Supplementary Files S1 and S2). In other words, critical reviews are necessarily subjective due to the interpretation needed for the appraisal, synthesis and analysis of the literature (Grant and Booth, 2009). For instance, one of the papers we excluded due to a lack of conceptual clarity distinguished an additionally coercive rationale (Bidwell and Schweizer, 2021), to reflect the critique that public involvement in research provides opportunities for powerful interests to manipulate the decision process and silence opposition. Although this coercive rationale was on average not endorsed by the US general public (Bidwell and Schweizer, 2021), its presence could be context-specific, and its added value thus worthwhile to explore further.

A final limitation lies in the broad scope of our critical review. We included publications on PPI in research and in policy and practice, from the domain of health promotion and from other domains, and from a period covering more than 30 years. However, we neither systematically compared the articulation of rationales from different contexts, domains and points in time, nor did we conduct an in-depth analysis of the participatory research practices that were covered by the publications included in our review. The number of empirical studies that explicitly make use of rationales in the evaluation of PPI in research seems too small to conduct such comparisons or in-depth analyses. Issues like these could be interesting to explore further once more eligible studies have become available.

### Interpretation

We have demonstrated the applicability of the five categories of rationales in a case study on PPI in Lyme research. Although outside the domain of health promotion, the applicability of different rationales for PPI in research has also been shown in three multiple case studies (Webler and Tuler, 2006; Bauer and Pregernig, 2013; Schmidt *et al.*, 2020). These revealed that rationales seldom become manifest in pure form (Bauer and Pregernig, 2013). First, different participants within one case can endorse different rationales (Webler and Tuler, 2006; Schmidt *et al.*, 2020). In the Lyme research case, for instance, we saw how the funder initiated the involvement of patients to do them justice (transformative), while the chair of the invitational conferences invited them in order to give them a voice (democratic). To give another example, in a study on transdisciplinary research projects, the funding agency expected stakeholder involvement to contribute to implementable outcomes (instrumental), while scientists thought that the best motivation for stakeholder engagement was knowledge integration (substantive; Schmidt *et al.*, 2020). These findings additionally imply that the extent to which different rationales are being endorsed within a single case may differ between research cases (Webler and Tuler, 2006). Second, the dominant rationale within cases may vary over time (Bauer and Pregernig, 2013; Schmidt *et al.*, 2020). In the Lyme research case, for example, we recognized knowledge integration (substantive) during the design of the research, while efficiency reasons (instrumental) tended to occur particularly during the conduct of the research. Another example stems from the study on transdisciplinary research: almost all project activities gradually also came to serve implementable outcomes (instrumental), while these activities initially followed other rationales (e.g. democratic or substantive; Schmidt *et al.*, 2020). These empirical findings highlight the presence of multiple rationales for PPI in research, which runs the risk of creating mismatches between the expectations of—and thus conflicts between—the different participants (Glimmerveen *et al.*, 2018). Hence, PPI is dynamic, and carrying out PPI is about managing the different underlying reasons that patients, public and other stakeholders may contribute. To allow for such management, the different rationales should be noted, explored and made explicit (Boote *et al.*, 2002; Knaapen and Lehoux, 2016). We feel we can claim that our categories of rationales may serve as a frame of reference in this respect.

Starting from the—potentially conflicting—means or ends they aim for (Knaapen and Lehoux, 2016), each rationale for PPI in research is expected to have its own implications for the design of the dimensions of the participatory process (Cousins, Whitmore, and Shulha, 2013). This dependence was illustrated in an analysis of participatory knowledge production practices, where different rationales (democratic, substantive and instrumental) led to different framings of the designation of the participants (citizens, lay persons or stakeholders), their selection (representativeness, expertise and background) and what they were expected to provide (representative values, inclusive knowledge or interests; Bauer and Pregernig, 2013). The example of the democratic rationale from our Lyme research case additionally shows the potential influence of the ‘participatory playing field’ on such dimensions. That is, ‘what’ may determine ‘who’ should be involved, such as, in our case study, involving the general public rather than patients in drafting the research agenda for the prevention of Lyme disease. These findings confirm previous hypotheses and earlier empirical insights that highlighted the importance of ‘intentionality’ for PPI (Stirling, 2006)—be it in research, policy or practice (Cornwall, 2008; Wesselink *et al.*, 2011; Ives *et al.*, 2013)—and the advisability of distinguishing between patients and the public in this respect (Fredriksson and Tritter, 2017). Therefore we agree that, in order to substantiate the potential of PPI in research, its underlying reasons should be made explicit from the start and be regularly reflected on during implementation (Wilson *et al.*, 2015; McCoy *et al.*, 2018; Schmidt *et al.*, 2020). We think that our overview of rationales may guide the anticipated discussions in this respect.

### Implications

Although it is not a panacea, our overview of rationales can offer added value for the implementation, evaluation and conceptualization of PPI in research. First, it may support implementation by taking it as the—currently lacking—starting point for the substantiation of the available indicators for successful PPI (McCoy *et al.*, 2018). This may prevent PPI from becoming ‘nominal’, meaning that it solely serves as window dressing (Wesselink *et al.*, 2011; Ball *et al.*, 2019), for instance, because it is started in response to conditions for research funding (Ball *et al.*, 2019). Such nominal PPI may be fairly prevalent, as one of the most cited challenges for PPI in research is the concern about tokenistic involvement (Domecq *et al.*, 2014).

Second, using our overview to clarify the rationales present in participatory research could support the joint development of an impact pathway hypothesis, in order to facilitate the implementation of PPI (Knaapen and Lehoux, 2016; Schmidt *et al.*, 2020). This may enhance the positive and avoid the negative impacts of PPI in research (Gradinger *et al.*, 2013) as well as provide better opportunities to assess its added value (Brett *et al.*, 2010). The fact that most previous evaluation studies of PPI in the research were found to be poorly theorized (Brett *et al.*, 2010; Mockford *et al.*, 2012; Nitsch *et al.*, 2013; Lander *et al.*, 2014), indicates that, at present, the greatest progress might be made by carefully conceptualizing PPI in future participatory research.

Third, using our overview could provide more coherence to the—so far fragmentary—concept of PPI in health promotion research (Nitsch *et al.*, 2013; Wilson *et al.*, 2015). If combined with dimensions of the participatory process (Cousins *et al.*, 2013; Nitsch *et al.*, 2013), evaluation studies recognizing different rationales could provide a better understanding of how to connect the *why* of PPI (McCoy *et al.*, 2018) with the type of patients and/or public to involve, how to involve them, in what, and what they are expected to bring (Cousins and Whitmore, 1998; Weaver and Cousins, 2007; Ives *et al.*, 2013; Domecq *et al.*, 2014; Fredriksson and Tritter, 2017).

## CONCLUSION

Our critical review yielded five conceptually different categories of rationales for PPI in research. Together, these may be used as a frame of reference to explore, make explicit and reflect on the different rationales in health promotion research. This might help to manage the dynamics of the participatory process, define realistic purposes, select matching approaches and design appropriate evaluation studies. These evaluations, in turn, may improve our understanding of how different rationales relate to the dimensions of the participatory process, and thus contribute to a more coherent conceptualization of PPI in research. Any effort to further extend or refine the categories of rationales we distinguished should preferably involve research groups from different domains.

## SUPPLEMENTARY MATERIAL

Supplementary material is available at *Health Promotion International* online.

## Supplementary Material

daac016_Supplementary_Data
