# Supplementary material for: Participatory research in health promotion: a critical review and illustration of rationales
Source: Health Promot Int. 2022 Jun 23;37(Suppl 2):ii7–ii20. doi: 10.1093/heapro/daac016 (PMC9226656; doi:10.1093/heapro/daac016)
Supplement: daac016_Supplementary_Data [file daac016_supplementary_data.zip › Suppl file I Excluded papers HPI REFnew.docx]

**Supplementary file I**

**Papers excluded from the thematic analysis and description of rationales for patient and public involvement in health promotion research**

Atkinson, J. A., Vallely, A., Fitzgerald, L., Whittaker, M., & Tanner, M. (2011). The architecture and effect of participation: a systematic review of community participation for communicable disease control and elimination. Implications for malaria elimination. *Malar J, 10*, 225. doi:10.1186/1475-2875-10-225

Bidwell, D., & Schweizer, P.-J. (2020). Public values and goals for public participation. *Environmental Policy and Governance, n/a*(n/a). doi:https://doi.org/10.1002/eet.1913

Blackstock, K. L., Kelly, G. J., & Horsey, B. L. (2007). Developing and applying a framework to evaluate participatory research for sustainability. *Ecological Economics, 60*(4), 726-742. doi:https://doi.org/10.1016/j.ecolecon.2006.05.014

Cousins, J. B., Whitmore, E., & Shulha, L. (2013). Arguments for a Common Set of Principles for Collaborative Inquiry in Evaluation. *American Journal of Evaluation, 34*. doi:DOI: 10.1177/1098214012464037

Cuppen, E. (2010). *Putting Perspectives into Participation. Constructive Conflict Methodology for problem structuring in stakeholder dialogues.* Free University of Amsterdam, Amsterdam.

De Zeeuw, C. (2005). *Unpacking rationales for community participation in urban regeneration in the Netherlands*. Paper presented at the Association of European Schools of Planning, Vienna.

Dean, R. J. (2017). Beyond radicalism and resignation: the competing logics for public participation in policy decisions. *Policy & Politics, 45*(2), 213-230. doi:10.1332/030557316X14531466517034

Farthing, R. (2012). Why youth participation? Some justifications and critiques of youth participation using New Labour's youth policies as a case study. *Youth & Policy, 109*, 71-97.

Glimmerveen, L., Ybema, S., & Nies, H. (2018). Empowering citizens or mining resources? The contested domain of citizen engagement in professional care services. *Soc Sci Med, 203*, 1-8. doi:10.1016/j.socscimed.2018.03.013

Kenny, A., Farmer, J., Dickson-Swift, V., & Hyett, N. (2014). Community participation for rural health: a review of challenges. *Health Expect*. doi:10.1111/hex.12314

Lindblom, S., Flink, M., Elf, M., Laska, A. C., von Koch, L., & Ytterberg, C. (2021). The manifestation of participation within a co-design process involving patients, significant others and health-care professionals. *Health Expect, 24*(3), 905-916. doi:10.1111/hex.13233

Madden, M., & Speed, E. (2017). Beware Zombies and Unicorns: Toward Critical Patient and Public Involvement in Health Research in a Neoliberal Context. *Frontiers in Sociology, 2*(7). doi:10.3389/fsoc.2017.00007

Martin, G. P. (2008). 'Ordinary people only': knowledge, representativeness, and the publics of public participation in healthcare. *Sociol Health Illn, 30*(1), 35-54. doi:10.1111/j.1467-9566.2007.01027.x

Nitsch, M., Waldherr, K., Denk, E., Griebler, U., Marent, B., & Forster, R. (2013). Participation by different stakeholders in participatory evaluation of health promotion: A literature review. *Evaluation and Program Planning, 40*, 42-54.

Reid, A., & Nikel, J. (2008). Differentiating and evaluating conceptions and examples of participation in environment-related learning. In A. Reid, B. B. Jensen, J. Nikel, & V. Simovska (Eds.), *Participation and learning* (pp. 32-59). Dordrecht: Springer.

Rifkin, S. B. (1996). Paradigms lost: toward a new understanding of community participation in health programmes. *Acta Tropica, 61*(2), 79-92.

Rifkin, S. B. (2014). Examining the links between community participation and health outcomes: a review of the literature. *Health Policy Plan, 29 Suppl 2*, ii98-106. doi:10.1093/heapol/czu076

Rowe, G., & Frewer, L. J. (2004). Evaluating public-participation excersises: A research agenda. *Science, Technology & Human Values, 29*(4), 512-556.

Suškevičs, M. (2019). Legitimate planning processes or informed decisions? Exploring public officials' rationales for participation in regional green infrastructure planning in Estonia. *Environmental Policy and Governance, 29*(2), 132-143. doi:https://doi.org/10.1002/eet.1836

Wagemakers, A., Corstjens, R., Koelen, M., L., V., Van 't Riet, H., & Dijkshoorn, H. (2008). Participatory approaches to promote healthy lifestyles among Turkish and Moroccan women in Amsterdam. *Promotion & Education, 15*, 17-23.
