# Supplementary material for: Participatory research in health promotion: a critical review and illustration of rationales
Source: Health Promot Int. 2022 Jun 23;37(Suppl 2):ii7–ii20. doi: 10.1093/heapro/daac016 (PMC9226656; doi:10.1093/heapro/daac016)
Supplement: daac016_Supplementary_Data [file daac016_supplementary_data.zip › Suppl file II Papers included HPI REFnew.docx]

**Supplementary file II**

**Papers included in the thematic analysis and description of rationales for patient and public involvement in health promotion research**

| **Author and year**  **Type of publication**  **Type of involvement** | **Rationales as typified in the articles** | **Rationales as categorized by the authors** |
| --- | --- | --- |
| **Involvement in research in the health domain** | | |
| **No. 1**  (Boote, Telford, & Cooper, 2002)  Review and research agenda with respect to consumer involvement in health research  Distinguishes two distinct discourses. Describes also four rationales, but does not identify these as such. | **Moralistic arguments**  Ethically right for consumers to have a voice in research which will (or may) have an impact on their health status | **Democratic** |
|  | **Value statements**  Consumers can have a positive impact on health research | Undefined |
|  | **Lay theories** should inform and shape the research question. Have a voice in formulation of research questions. Ensure that study will answer questions that lay persons consider important. Promotes reliable, relevant research of importance to patients and those caring for them. Producing new knowledge by systematic inquiry with the collaboration of those affected by the issue being studied. | **Substantive** |
|  | **Recruiting** peers to research projects to access more marginalized members of society. **Disseminate** research information to peers. | **Instrumental** |
|  | **Empowerment** perspective related to issues of control and accountability. It has the potential to strengthen disadvantaged or disempowered groups by giving them the chance to speak out on research issues. Contribute to the practical concerns of people in an immediate problematic situation and to the goals of social science by joint collaboration within a mutually acceptable ethical framework, i.e. to negotiate a balance between developing valid generalizable knowledge and benefiting the community that is being researched. | **Transformative**  Including democratic element |
|  | **Consumer** perspective related to issue of rights, value for money, economic rights, satisfaction | **Neo-liberal** |
| **No. 2**  (Gradinger et al., 2013)  Narrative review of values associated with public involvement in health and social care research.  Distinguishes two overarching value systems with respect to rationales for involvement. | **Normative** value system, focused on moral, ethical and political concerns. Including:  **Empowerment**: transfer of control, self-help, seeking to overcome discrimination and oppression.  **Rights**: being of intrinsic value, about the fundamental human right to have a say.  **Change/action**: generating or translating knowledge into action in order to incite change.  **Accountability/transparency**: public accountability and transparency about research and public involvement.  **Ethical** values: ethical awareness in order to protect from harm. | **Democratic**  **& Transformative** |
|  | **Substantive** value system: focused on the concerns about the consequences of public involvement in research.  **Effectiveness**: to have an effect on research and implementation.  **Quality-relevance**: increasing quality, relevance, appropriateness and credibility of research.  **Validity/reliability**: processing reliable, valid and rigorous knowledge  **Representativeness/objectivity/ generalizability**: creating representative, objective and generalizable knowledge.  **Evidence**: generating substantial, consistent, comparable and replicable evidence-base about public involvement. | **Instrumental**  **& Substantive**  Including democratic elements |
| **No. 3**  (Ives, Damery, & Redwod, 2013)  Ethical reflection on moral and pragmatic motivations for patient and public involvement  Identifies two sets of motivations for patient and public involvement in health research | **Ideological, rights based**  Draws on broader social and ethical narratives around democratic representation of community values and preferences, transparency, accountability and responsibility. Also aiming at equalizing power imbalance between the public and the academic community. | **Democratic**  Including transformative elements |
|  | **Pragmatic and outcome oriented**  Involvement as a means to an end. Involvement positively impacts on the quality of the research processes and outputs, and promotes more reliable, relevant research. Adds insights to the design, methods and findings. Assists in dissemination and implementation. | **Substantive**  **& Instrumental** |
| **No. 4**  (Ball, Harshfield, Carpenter, Bertscher, & Marjanovic, 2019)  Literature review on patient and public involvement and engagement in research.  Distinguishes two types of motivations for researchers to integrate patient and public involvement in their projects. | **Pragmatic incentives**  **Condition** for research funding.  **A response** to a policy imperative to share power and control over research between the research community and the wider public.  **A practical way** of helping with the recruitment for and retention of participants in studies, and for securing acceptability of the study. | **Nominal**  **& Instrumental** |
|  | **Values, experiences and perceptions**  Believing it is **the right thing to do** and related to a moral dimension.  Belief that it will improve **research outputs**, for example to optimize clinical tools developed through research.  **Prior experiences**, like feeling that it improved the quality or relevance of a prior research that researchers were engaged in. | **Democratic**  **& Instrumental**  **& Substantive** |
| **No. 5**  (McCarron et al., In press 2021)  Scoping review of patients as partners in health research, including a thematic analysis of the purposes of engagement.  Distinguishes five themes. | Documenting and **advancing patient and public involvement** including goals of advancing the science of patient involvement | **Democratic**  **& Transformative** |
|  | **Relevance of research**: making research more meaningful to those affected.  **Impact on research**: through engagement in both conceptual aspects, such as defining and refining the research scope and questions, and practical elements, like participant recruitment | **Substantive**  **& Instrumental** |
|  | **Co-building**: involving individuals in the development of a framework, tool or strategy | **Instrumental** |
|  | **Capacity building**: creating opportunities to build the skills, confidence and knowledge of patients. | **Transformative** |
| **Involvement in research other than in health domain** | | |
| **No. 6**  (Cousins & Whitmore, 1998; Weaver & Cousins, 2007)  Literature reviews aimed at unpacking the framing participatory evaluation and the participatory process, respectively.  Distinguish two and three different justifications for collaborative social inquiries, respectively. | **Transformative participatory evaluation**  Political justification for collaborative inquiry. Rooted in normative conceptions of social justice and the democratic process. Primary interest is to promote fairness among all groups with a stake. Providing opportunities to have voice for oppressed or marginalized groups. Emancipatory though focus on the amelioration of social inequities. | **Democratic**  **& Transformative** |
|  | **Practical-participative evaluation**  Pragmatic justification for collaborative inquiry. Purported to lead to instrumental consequences and to increase the usefulness of the knowledge that is created. Takes on a problem-solving orientation and produces knowledge that bears upon identifiable practical problems. | **Instrumental**  Including substantive elements |
|  | **Stakeholder-based evaluation**  Epistemological justification for collaborative inquiry. Primary aim is the production of valid knowledge or representations of underlying social phenomena. Epitomizes the concept of local knowledge and the importance of context. Constructivist conception of research. | **Substantive** |
| **No. 7**  (Webler & Tuler, 2006)  Multiple-case study with quantitative assessment of perceptions on public participation processes in environmental decision making.  Identifying four perspectives on participation. | **Efficient cooperation**  Central belief that the purpose is to give recommendations to the responsible agency which will then make a decision which should serve a collective good. | **Instrumental**  Including democratic elements |
|  | **Egalitarian deliberation**  Emphasizes the importance of empowering participants and is a reaction against dominance of the agency over the process. | **Transformative** |
|  | **Science-centered stakeholder consultation**  Decision making is streamlined and task-centered, with a clear utilitarian focus on producing real progress on the problem rather than producing advice for sponsoring agency. | **Instrumental**  Including substantive elements |
|  | **Informed collaboration**  Ends-oriented process that makes progress on central problems and which provides legitimacy for the agency to act, through high quality information & establishment of trust. | **Instrumental** |
| **No. 8**  (Mielke, Vermaßen, Ellenbeck, Fernandez Milan, & Jaeger, 2016)  A critical review of stakeholder involvement in sustainability science and transformative science.  Distinguishes four types of stakeholder involvement in opening up science to society, based on the type of stakeholders involved. | **Democratic**  Stakeholder dialogues have the objective to integrate actors that are touched by the issue under consideration. Includes all stakeholders affected, including citizen initiatives. | **Democratic** |
|  | **Functionalist**  Irritating the science system with other social perspectives and relevance criteria in order to make science more sensitive for societal problems and to increase the probability that changes take place. Includes powerful stakeholders from all social sub-systems. | **Instrumental**  Including substantive elements |
|  | **Technocratic type**  Improving the research process by broadening the extent of available information. Includes technical experts, like planners, engineers, other scientists. Does not include citizens. | **Substantive** |
|  | **Neo-liberal-rational**  A tool for both groups in science-society interfaces to impose their perceptions and interests on each others. Includes stakeholders with interests, such as lobby groups advocating their individual, organizational or political interests. | **Neo-liberal** |
| **No. 9**  (Schmidt, Falk, Siegmund-Schultze, & Spangenberg, 2020)  Literature review on objectives of stakeholder involvement in transdisciplinary research.  Conceptual framework which includes four rationales for non-academic stakeholder involvement.  Type of stakeholders not further specified. | **Normative**  People should be given the opportunity to contribute to the process of knowledge generation that affects them. Those who are affected by or can affect the problem under investigation, should have a voice when formulating, conducting and implementing research, like in refining the research question and implementing its process and outcomes. | **Democratic**  Including substantive elements |
|  | **Social-learning**  Stimulating processes of social learning to understand and solve the problem. Empowerment by giving those marginalized voice. Awareness-raising and improving mutual understanding about conflicting interests, potential conflicts, values and capacities Considering one another’s needs, norms and visions, which may guide transformative processes. Enabling new networks, building of trust and identifying balanced solutions. May eventually contribute to lasting behavior change. | **Transformative**  Including instrumental elements |
|  | **Substantive**  Improvement of the quality and significance of research. The exchange between and integration of the various bodies of knowledge, perspectives and approaches in order to co-produce a socially robust, holistic and shared understanding of problems and objectives, as to increase the context-specific relevance and to identify locally adapted solutions. Scientific knowledge is just one of many legitimate bodies of knowledge. | **Substantive** |
|  | **Implementation**  Increase of acceptance and legitimacy of the process outcomes and aspired-to solution of the studied problem. Engagement builds common ground and trust, which increased motivation and commitment to contribute to a joint process. Giving voice and interest leads to growing acceptance, legitimacy and ownership of process and outcomes. Increases motivation and capacity to implement new knowledge and the impact and long-term usage of that knowledge. | **Instrumental**  Including democratic elements  Including transformative elements |
| **Involvement in services, programs and/or policy in health domain** | | |
| **No. 10**  (Oakley, 1989)  An examination of critical issues with respect to community involvement in health development.  Summary of arguments, set forward by different studies, for adopting community involvement as a strategy for community development. | **Basic right**  Involvement is basic right which all people should be able to enjoy. Involvement in the decisions that affect people’s health, builds self-esteem and sense of responsibility. Involvement is of intrinsic value in development of communities in a wider sense. | **Democratic**  Including transformative elements |
|  | **Political consciousness**  Involvement makes people aware that they could become usefully involved in development in general. Breaks the knot of dependence in health development work. Makes people politically conscious and eager to make their voice heard in other development processes. | **Transformative** |
|  | **Effectiveness**  Involvement is appropriate and successful in meeting health needs as defined by local people, as opposed to medical needs defined by health authorities. Takes into account local perceptions of health needs. Programs managed with the support of local people have a better chance of being successful | **Instrumental**  Including substantive elements |
|  | **Efficiency**  Involvement as a means to make more resources available by drawing upon local knowledge and resources to complement formal health services.  Can help to extend coverage of services and to lower overall costs. May increase long-term cost-effectiveness through -term adequate return on investment. | **Instrumental** |
| **No. 11**  (Charles & DeMaio, 1993)  Conceptual framework for lay participation in health care decision making. | **Valued as an end in itself**  Lay participation has intrinsic value. The issue of representativeness is crucial because the opportunity to participate in decision making is highly valued. The extent to which the views of all affected persons or groups are represented in the assessment and selection of alternative options will be of key importance. | **Democratic** |
|  | **Valued as a means**  Participation is a means to achieving other policy goals. It should be evaluated according to whether it helps achieve these goals. Its merits in health care decision-making should not be assumed; rather, the extent to which stated goals are achieved is an empirical question requiring further research. | **Instrumental** |
| **No. 12**  (Morgan, 2001)  Literature review of trends in participation literature.  Distinguished two definitional models of community participation. | **Empowerment tool**  Involvement as empowerment tool through with communities take responsibility for diagnosing and working to solve their own health and development problems. Participation as an end. | **Transformative** |
|  | **Utilitarian effort**  Involvement as utilitarian tool as to use community resources to offset the costs of providing services. Participation as e means to accomplish the aims of a project more efficiently, effectively or cheaply. | **Instrumental** |
| **No. 13**  (Litva et al., 2002)  Empirical study on impulses towards public involvement in health care decision making.  These four impulses to participation were described previously by (Parry, Moyser, & Day, 1992). | **Communitarian**  Involvement relates to the common interests of a particular community. with the assumption that the expertise of the public should lead to more appropriate provision of local health services. | **Democratic**  Including substantive elements |
|  | **Instrumentalist**  Participation should promote or defend the goals of the participants; with the minimum of costs and the maximum of effects. It is essentially self-interested and in health care particularly linked to participation among users. | **Instrumental**  Including democratic elements  Including consumerist elements |
|  | **Educative**  Participation is concerned with the development of citizen senses of competence and responsibility, with the concern to enhance democracy by encouraging citizens to reach their full potential which links to expressive. Creates a more participatory society, moral influence, as people come to appreciate more fully the interests and aspirations of their fellows, society becomes more integrated and trusted | **Transformative**  Including democratic elements |
|  | **Expressive**  Participation as an expression of their political identity and belonging. People do not act in the expectation of directly achieving a goal or out of a concern for their community, rather to express their feelings or to display their stance about a matter. At its broadest form, this may be a form of symbolic participation. | **Transformative**  Including nominal elements |
| **No. 14**  (Tritter & McCallum, 2006)  A critical, empirical appraisal of power in user involvement in health services.  Distinguishes three theoretical justifications for user involvement. | **Local democracy-accountability**  Relates to rights inherent in citizenship. Involvement of voters. Seeks to ensure that current user’s views are not prioritized over other residents who may be future service users. Aiming at local accountability or engagement with the spectrum of the users. Distinguishes direct and active user involvement from indirect and passive involvement. | **Democratic** |
|  | **Citizen-taxpayers’ fund public health services**  Relates to the rights of individual choice in the market place. Involvement of taxpayers. Neo-liberal value for money justification to strengthen the public’s voice in decisions about the organization and delivery of health services. Requires empowered staff to be responsive to the expressed needs of users. Shift from patient involvement to patient and citizen involvement recognizes that most residents remain taxpayers and potential patients | **Neo-liberal** |
|  | **Consumerism-informed consent**  Relates to the rights of individual choice in the market place. Involvement for consumers. Central to neo-liberal approaches to realizing health services. Promoting a patient choice agenda redefines focus of health service provision and reframes healthcare providers as vendors. User involvement as feedback mechanism for the expression of consumer views. It is an essential component of markets. Promotes patient-focused care and redresses power inequalities between health professionals and patients. Yet, consumerism and individual choice is more about customer relations than any enhanced rights which entail true partnership or power sharing. | **Neo-liberal** |
| **No. 15**  Anonymized  Literature review on the aims of participation in in health promotion.  Distinguishes three anticipated outcomes of participation. | **Instrumental**  Participation supports the provision of health promotion programs. Makes programs more transparent and acceptable, creates more community support. Makes programs more effective through improved performance and offering higher quality. Makes programs more efficient through increased reach among community members and ore cost-effectiveness. | **Instrumental** |
|  | **Empowerment (individual)**  Participation benefits those individual who participate with spin-off to their community. Increased responsibility through higher commitment and more sensible choices. Self-fulfillment through skills development, self-efficacy. Participants gain greater control over decisions that affect their life. | **Transformative**  Including democratic elements |
|  | **Empowerment (collective)**  Community as a whole gains strength and acquires delegated or shared responsibility. Strengthened of health promotion efforts, social structures and networks, and mutual support. Community gains greater control over policy and political influence. | **Transformative**  Including democratic elements |
| **No. 16**  (Preston, Waugh, Larkins, & Taylor, 2010; Taylor, Wilkinson, & Cheers, 2008)  Community participation in health services development.  Distinguish four conceptual approaches for participation. | **Development**  Local people, in partnership with professionals, have a role in decision making and in achieving the outcomes they consider are important. Is underpinned by the principles of social justice. Conceptualizes health and social care development as interactive, evolutionary process, embedded in a community of interest. | **Transformative**  Including neo-liberal elements  Including democratic elements |
|  | **Community empowerment**  Seeks to empower and support communities, individuals and groups to take greater control over issues that affect their health and wellbeing. Includes notions of personal development, consciousness raising and social action. | **Transformative** |
|  | **Instrumental**  Defines health and wellbeing as an end result, rather than as a process, with participation as an intervention supporting other public health or primary health care interventions, health planning or service development. Usually led by professionals and important components of services are predetermined according to local and national priorities. | **Instrumental** |
|  | **Contributions**  Considers participation primarily as voluntary contributions, to a project, such as time, resources or community-based knowledge. Participation usually led by external professional developers who make decisions about how contributions will be used. | **Instrumental**  Including nominal elements |
| **No. 17**  (Conklin, Morris, & Nolte, 2010)  Literature review on the conceptual and empirical evidence for public involvement in healthcare policy.  Distinguishes four perspectives on public involvement in healthcare policy. | **Democratic perspective**  Assumes that involvement is good thing either in principle or for instrumental purpose. Expected to positively impact on public decisions or to protect citizens from others making decisions against their interests. Includes republican or communitarian position where participation is viewed as social obligation; as duty to engage actively in constructing or shaping the community. | **Democratic**  **& Instrumental**  Including transformative elements |
|  | **Development perspective**  Involvement regarded either as opportunity to express political and belonging, or as educative. The latter includes increasing both the citizens’ capability and confidence for political engagement, their understanding the challenges of policy making, and their knowledge of their conditions and services. | **Transformative** |
|  | **Consumerist perspective**  Assumes that the public as consumers or customers of healthcare have the right to demand services how they want them and that the expression of this demand will influence outcomes. Preferences are viewed as the lever to enhance responsiveness of service providers. No social action is implied beyond exercising individual choice as expression of demand. | **Neo-liberal**  Including instrumental elements |
|  | **Critical perspective**  Questions the authenticity and ultimate purpose of involvement. Argues that is may be viewed as a strategy that legitimates disagreeable change, allowing politicians to deflect criticism by suggesting broader consensus. | **Nominal** |
| **No. 18**  (Knaapen & Lehoux, 2016)  Conceptual models of patient and public involvement in setting standards for health care.  Categorizes arguments in favor of patient and public involvement into three main types. | **Democratic voice model**  The rights of citizens to democratic decision making on a policy or collective level. Premised on notions of individual rights, civic responsibility, social justice, and political accountability. May include (potential) users and recipients of services as well as taxpayers (as financial stakeholders). | **Democratic**  Including transformative elements  Including neo-liberal elements |
|  | **Consumer choice model**  Individual consumer rights in health care. Includes access to full information and freedom to choose (i.e. patients’ autonomy). Involving autonomous consumers contributes to personalizing clinical care, which may include personal preferences and decision-making. | **Neo-liberal** |
|  | **Lay expertise model**  Lay participants contributing to specific expertise. Experiential knowledge from those affected improves health research. Considers guidelines as knowledge tools. | **Substantive**  Including instrumental elements |
| **Involvement in services, program and/or policy in domains other than health** | | |
| **No. 19**  (Cornwall, 2008; White, 1996)  Aimed to distinguish the different interests that public involvement me serve in community development.  Distinguish four rationales for public involvement. | **Representative**  Participation means sustainability, and to avoid creating dependency, for the organizing agency. For participants it may mean leverage, to influence the shape the project and its management takes. People are given voice in determining their own development. | **Democratic** |
|  | **Transformative**  Being involved in considering options, making decisions and taking collective action to fight injustice is empowering itself. For the implementing agency, participation means enabling people to make their own decisions, work out what to do and take action. For participants it means empowerment: to decide and act for themselves. May serve both as a means and an end, a continuing dynamic. | **Transformative** |
|  | **Instrumental**  Participation means efficiency to the implementing agency, to make projects more cost-effective, which may bring costs for the target group (e.g., time spent). | **Instrumental** |
|  | **Nominal**  Participation means legitimation to the implementing agency to show they are doing something (display). Existence of public involvement demonstrates that an agency is ‘doing something’ and ‘has a popular base’, which may be significant in claims for extra personnel or financial support. Still, for participants, nominal involvement may mean inclusion and access to potential benefits. | **Nominal**  Including transformative elements |
| **No. 20**  (Stirling, 2006, 2008)  Conceptual basis for participatory deliberation on the cutting edge of “appraisal” and “commitment” in technology choice.  Distinguishes three imperatives in appraisal that are all susceptible to instrumental framing. | **Normative considerations**  Based on considerations of democratic principle, participation is regarded as an end in and of itself. A participatory process is a self-evidently good thing, without reference to the ends in question. An intrinsic social desirability of equity of access, empowerment of process and equality of outcomes. | **Democratic**  Including transformative elements |
|  | **Substantive reasons**  Participation increases the breadth and/or depth of the information underlying decision making. Through gathering more diverse, extensive and context-specific bodies of knowledge, and taking more careful and explicit into account the divergent values and interests, participation enhancing the ‘quality’ of the decisions themselves. Hence, participation serves as a means to an end. Ostensibly blind to political power. | **Substantive** |
|  | **Instrumental** **imperatives**  Participation regarded in terms of efficacy in realizing particular favorite ends, with little tangible impact on policy making. May aim to sustain or restore public credibility and trust, and more effective decision justification. Participation serves as a means to an end. | **Instrumental**  Including nominal elements |
| **No. 21**  (Wesselink, Paavola, Fritsch, & Renn, 2011)  Practitioners’ perspectives on rationales for participation in environmental policy.  Empirical research based on the conceptualization by (Stirling, 2006, 2008). | **Normative**  Democratic ideals call for maximum participation. It aims to counter the power of incumbent interests and allows all who are affected by a decision to have influence. | **Democratic** |
|  | **Substantive**  Participation aims to increase the breadth and depth of information and thereby improve the quality of decisions. Non-experts see problems, issues and solutions that experts miss. It ignores power issues such related to problem framing. Unlike in the instrumental rationale, policy goals can be changed in a substantive rationale. | **Substantive** |
|  | **Instrumental**  Participation makes decisions more legitimate and improves results. It aims to restore public credibility, diffuse conflicts, justify decisions and to limit future challenges to implementation by ‘creating ownership’. Policy goals are not open for discussion, only the details are (to a lesser or greater extent). It hereby supports incumbent interests. | **Instrumental** |
|  | **Legalistic**  Participation occurs only because it is required, as a formality, to meet formal requirements, and organized due to procedural pressure, like from dictates of a central authority as a major reason to start public involvement. Compliance with such pressure or rules is necessary to get things done, without any uptake of results. | **Nominal** |
| **No. 22**  (Head, 2011)  Conceptual framework for understanding the various forms of youth participation and the associated rationales. | **Rights**  Rights based legal principles, when translated into regulatory form, can impose legal duties to consult young people in certain circumstances, which can underpin moral respect. | **Democratic** |
|  | **Developmental benefits**  Individual and wider social benefits are expected from the experience of being engaged, such as skills, self-esteem, self-development, and broadening civic activity and contributions to citizenship. | **Transformative** |
|  | **Efficiency and better services**  Services, programs and policies will become more efficient and effective if young people’s perspectives are angaged. | **Instrumental** |
| **No. 23**  (Bauer & Pregernig, 2013)  Examination of the conceptualization and realization of participation by identifying their underlying rationales.  Distinguishes three rationales in ten technology assessment and foresight projects. | **Political rationale**  Participation driven by desire to ensure consensus and increase the acceptance and legitimacy of the results of the participatory process. The concept of citizens stresses the democratic quality of the political rationale. The involvement of different actor groups holds the promise of providing more weight and legitimacy to future-oriented knowledge. Increased social robustness of visions and scenario’s, that are also more easily pursued through consensus. | **Democratic**  Including substantive elements  Including instrumental elements |
|  | **Cognitive rationale**  Participants are expected to bring specific knowledge, but in a rather instrumental way, that is: to bring all relevant information, secure a variety of perspectives, and in the end, guarantee the reliability of the results. May include both ‘scientists’, ‘experts’ and ‘laypersons’. | **Substantive**  **& Instrumental** |
|  | **Constructive rationale**  Participation focusses more on enabling and self-reinforcing dynamics, as a means of learning, coordination and capacity-building. Participatory processes contribute in the long term to the realization of shared goals and visions. Contributing to improved coherence, in line with the development of a shared mental framework. Participatory processes align expectations and thus create a self-fulfilling prophecy. Relevant actors are defined as those who have an important role to play, although ‘stakeholders’ are much more important than ‘experts’ and ‘laypersons’. | **Instrumental**  Including transformative elements |

**References**

Ball, S., Harshfield, A., Carpenter, A., Bertscher, A., & Marjanovic, S. (2019). *Patient and public involvement in research. Enabling meaningful contributions*. Retrieved from Santa Monica, Calif., and Cambridge, UK:

Bauer, A., & Pregernig, M. (2013). Whose look into the future? Participation in technology assessment and foresight. *Critical Policy Studies, 7*(1), 18-36. doi:10.1080/19460171.2012.745992

Boote, J., Telford, R., & Cooper, C. (2002). Consumer involvement in health research: a review and research agenda. *Health Policy, 61*(2), 213-236.

Charles, C., & DeMaio, S. (1993). Lay participation in health care decision making: a conceptual framework. *J Health Polit Policy Law, 18*(4), 881-904. doi:10.1215/03616878-18-4-881

Conklin, A., Morris, Z. S., & Nolte, E. (2010). *Involving the public in health care policy. An update of the research evidence and proposed evaluation framework*. Retrieved from Santa Monica CA:

Cornwall, A. (2008). Unpacking ‘Participation’: models, meanings and practices. *Community development journal, 43*(3), 269-283.

Cousins, J. B., & Whitmore, E. (1998). Framing participatory evaluation. In E. Whitmore (Ed.), *Understanding and practicing participatory evaluation* (pp. 5-23). San Francisco, CA: Jossey-Bass.

Anonymized

Gradinger, F., Britten, N., Wyatt, K., Froggatt, K., Gibson, A., Jacoby, A., . . . Popay, J. (2013). Values associated with public involvement in health and social care research: a narrative review. *Health Expect, 18*(5), 661-675. doi:10.1111/hex.12158

Head, B. W. (2011). Why not ask them? Mapping and promoting youth participation. *Children and Youth Services Review, 33*, 541-547.

Ives, J., Damery, S., & Redwod, S. (2013). PPI, paradoxes and Plato: who's sailing the ship? *J Med Ethics, 39*(3), 181-185. doi:10.1136/medethics-2011-100150

Knaapen, L., & Lehoux, P. (2016). Three Conceptual Models of Patient and Public Involvement in Standard-setting: From Abstract Principles to Complex Practice. *Science as Culture, 25*(2), 239-263. doi:10.1080/09505431.2015.1125875

Litva, A., Coast, J., Donovan, J., Eyles, J., Shepherd, M., Tacchi, J., . . . Morgan, K. (2002). 'The public is too subjective': public involvement at different levels of health-care decision making. *Soc Sci Med, 54*(12), 1825-1837.

McCarron, T. L., Clement, F., Rasiah, J., Moran, C., Moffat, K., Gonzalez, A., . . . Santana, M. (In press 2021). Patients as partners in health research: A scoping review. *Health Expectations, n/a*(n/a). doi:https://doi.org/10.1111/hex.13272

Mielke, J., Vermaßen, H., Ellenbeck, S., Fernandez Milan, B., & Jaeger, C. (2016). Stakeholder involvement in sustainability science—A critical view. *Energy Research & Social Science, 17*, 71-81. doi:https://doi.org/10.1016/j.erss.2016.04.001

Morgan, L. M. (2001). Community participation in health: perpetual allure, persistent challenge. *Health Policy Plan, 16*(3), 221-230.

Oakley, P. (1989). *Community involvment in health development. An examination of the critical issues*. Geneva: World Health Organisation.

Parry, G., Moyser, G., & Day, N. (1992). *Political participation and democracy in Britain*. Cambridge: Cambridge University Press.

Preston, R., Waugh, H., Larkins, S., & Taylor, J. (2010). Community participation in rural primary health care: intervention or approach? *Aust J Prim Health, 16*(1), 4-16. doi:10.1071/py09053

Schmidt, L., Falk, T., Siegmund-Schultze, M., & Spangenberg, J. H. (2020). The Objectives of Stakeholder Involvement in Transdisciplinary Research. A Conceptual Framework for a Reflective and Reflexive Practise. *Ecological Economics, 176*, 106751. doi:https://doi.org/10.1016/j.ecolecon.2020.106751

Stirling, A. (2006). Analysis, participation and power: justification and closure in participatory multi-criteria analysis. *Land use policy, 23*(1), 95-107.

Stirling, A. (2008). “Opening up” and “closing down” power, participation, and pluralism in the social appraisal of technology. *Science, Technology, & Human Values, 33*(2), 262-294.

Taylor, J., Wilkinson, D., & Cheers, B. (2008). *Working with communities in health and human services*. Soth Melbourne: Oxford University Press.

Tritter, J. Q., & McCallum, A. (2006). The snakes and ladders of user involvement: Moving beyond Arnstein. *Health Policy, 76*(2), 156-168. doi:10.1016/j.healthpol.2005.05.008

Weaver, L., & Cousins, J. B. (2007). Unpacking the participatory process. *Journal of Multidisciplinary evaluation, 1*(1), 19-40.

Webler, T., & Tuler, S. (2006). Four Perspectives on Public Participation Process in Environmental Assessment and Decision Making: Combined Results from 10 Case Studies. *Policy Studies Journal, 34*(4), 699-722. doi:https://doi.org/10.1111/j.1541-0072.2006.00198.x

Wesselink, A., Paavola, J., Fritsch, O., & Renn, O. (2011). Rationales for public participation in environmental policy and governance: practitioners' perspectives. *Environment and Planning A, 43*(11), 2688-2704.

White, S. C. (1996). Depoliticising development: the uses and abuses of participation. *Development in practice, 6*(1), 6-15.
